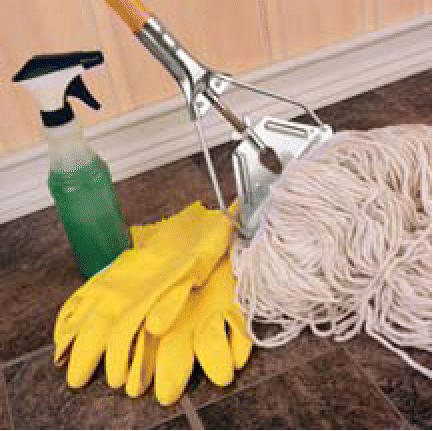# The Beat

**Published:** 2006-03

**Authors:** Erin E. Dooley

## Supersized Sun Power

At the end of 2004, the United States had 397 megawatts of solar energy capacity. Now two Southern California utility companies plan to harness the state’s abundant sunshine for two solar-powered plants that will produce more electricity than all of those solar energy projects combined. The new plants will use 40-foot dishes to focus the sun’s energy onto Stirling engines, sealed systems filled with hydrogen that, when heated with the solar energy, drive four pistons. A 500-megawatt power plant of 20,000 dishes will be located in the Mojave Desert, while a 300-megawatt plant of 12,000 dishes will call the Imperial Valley home. Construction will begin on the sites in 2008.

## Green Buses for Beijing Olympics

Beijing has signed a contract to replace 7,277 older city buses with new ones that meet higher environmental standards as part of its plan to host a “green” Olympic Games in 2008. The November 2005 decision is also in line with the city’s effort to combat heavy air pollution, which, due to temperature inversions, hangs in a cloud over the metropolis for three months of the year. Currently 17,507 buses operate in Beijing, and 4.36 billion person-rides were reported in 2004, making bus travel the city’s most popular mode of transport. The new buses will meet stringent Euro III standards for carbon monoxide, smoke, particulate matter, nitrogen oxide, and hydrocarbon emissions.

## West Africa Adopts Fishing Plan

Fish currently provides nearly a quarter of the protein in the African diet, yet sub-Saharan Africa is the only region in which the per-capita availability of fish is declining. To help ensure the security of this food source, government officials meeting at the August 2005 Secretariat of the Economic Community of West African States endorsed the Abuja Declaration on Sustainable Fisheries and Aquaculture in Africa. The declaration includes a five-point action plan to support capture fisheries, develop aquaculture, improve fish market chains, increase the benefits from the fish trade, and support government decision makers with information. Speaking at the meeting, Nigerian president Olusegun Obasanjo pointed out that for Africa’s fish consumption to remain at its already-low present level, fish production must increase by more than 250% by the year 2015.

## International Year of Deserts

Arid land covers one-third of the Earth’s surface. Each day, more arable land is lost to advancing deserts and more people fall victim to drought. In recognition of these problems, the UN has declared 2006 the International Year of Deserts and Desert-ification. Planned activities, including a week-long film festival, will explore ways to protect the biological diversity of areas affected by desertification as well as the knowledge and traditions of the 2 billion people living in those areas. The UN Convention to Combat Desertification reports that desertification and drought shave $42 billion off agricultural production each year. The resulting food insecurity, famine, and poverty foster social, economic, and political tensions that perpetuate the cycle of degradation.

## Mercury Warnings Go Multilingual

In November 2005, the San Francisco Board of Supervisors unanimously passed an ordinance requiring that markets and restaurants post warnings in English, Chinese, and Spanish that certain fish may contain harmful levels of mercury. The measure is authorized under California’s Proposition 65, which requires that consumers be warned of toxicants in the products they buy; it is the first to mandate multilingual warnings. Mercury is a potent neurotoxicant, and fetuses and young children are particularly sensitive to its effects. Studies have demonstrated that low-level exposures are linked with small changes in learning and intelligence.

## Detoxifying Dust Bunnies

Nearly one-fourth of the housing units in the United States have significant levels of lead present in dust, soil, or paint. Now researchers at the Saint Louis University School of Public Health report in the 15 January 2006 issue of *Environmental Science & Technology* that all-purpose floor and counter detergents remove lead-bearing dust from wood, wallpaper, and vinyl flooring as effectively as detergents developed specifically for removing lead. These findings contradict earlier recommendations that only lead-specific cleaners or high-phosphate detergents are effective for this purpose. According to lead author Roger Lewis, these findings will be incorporated into new HUD guidelines to be released in 2006.

## Figures and Tables

**Figure:**
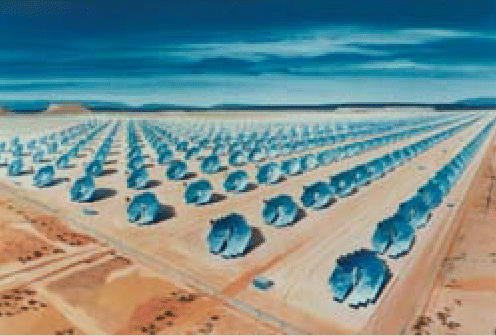


**Figure:**
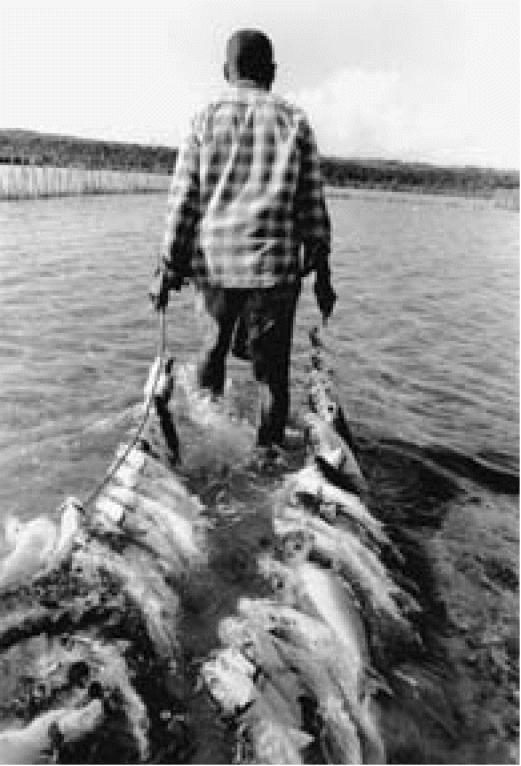


**Figure:**
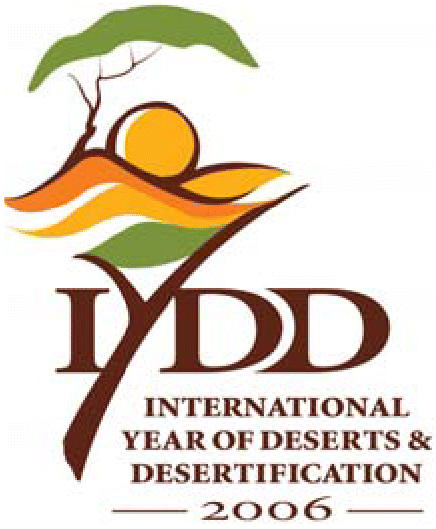


**Figure:**